# Comparison of the Analgesic Effects of Ultrasound-Guided Caudal Versus Ilioinguinal/Iliohypogastric Nerve Block Techniques for Pediatric Inguinal Surgeries: An Exploratory Randomized Controlled Study

**DOI:** 10.7759/cureus.85677

**Published:** 2025-06-10

**Authors:** Jyoti Burad, Amitabh Sen, Bhadrinath Narayanan, Fatma Al Shuhoumi, Safiya Hashim Al Hashimi, AbdulMajeed Al Balushi, Khadija Al Yaqoubi, Sachin Jose, Zainab Al Balushi

**Affiliations:** 1 Anesthesia and Intensive Care, Sultan Qaboos University Hospital, Muscat, OMN; 2 Biostatistics, Research Department, Oman Medical Specialty Board, Muscat, OMN; 3 Surgery, Sultan Qaboos University Hospital, Muscat, OMN

**Keywords:** flacc score, inguinal surgery, rescue analgesia, ultrasound-guided caudal block, ultrasound-guided lioinguinal/iliohypogastric nerve block

## Abstract

Background: Caudal nerve blocks are commonly used for inguinal surgeries in children but can lead to complications, such as prolonged weakness. The ilioinguinal/iliohypogastric (ILIH) nerve block is an alternative that is used less frequently. This study aimed to compare the effectiveness of these techniques in pediatric patients (ages six months to 12 years) undergoing inguinal surgery.

Methods: A double-blinded, randomized controlled trial was conducted with 20 pediatric patients undergoing inguinal surgery under general anesthesia. Group 1 (n = 8) received a caudal block, while Group 2 (n = 12) received an ILIH block. Pain was assessed using the Face, Legs, Activity, Cry and Consolability (FLACC) and Numeric Rating score (NRS). The primary outcomes included pain reduction and the use of rescue analgesia.

Results: The mean age of Group 1 was 2.13 years (SD=1.356), while that of Group 2 was 3.67 years (SD=1.303). The pain scores were 1.75 (SD=2.235) and 1.25 (SD=1.658) (p=0.792) at 30 minutes post-surgery in the Post Anesthesia Care Unit (PACU), 0.50 (SD=0.756) versus 0.08 (SD=0.289) (p=0.792) at PACU discharge, a score of zero versus 0.58 (SD=0.900) (p=0.792) at two hours post-operation for Group 1 and Group 2 respectively. Pain scores were similar at three hours, six hours, and at ward discharge. On day two at home, Group 1 reported a score of 0.25 (SD=0.707) compared to 0.33 (SD=0.778) in Group 2 (p=0.792). The use of rescue analgesia was higher in the caudal group, but the difference was statistically insignificant (p=0.7055). The block to ward discharge time was similar: 283 (SD=102.182) minutes versus 309.83 (SD=79.409) minutes for Group 1 and Group 2, respectively (p=0.746).

Conclusion: Both caudal and ILIH blocks provided similar analgesic effects for pediatric inguinal surgery. More extensive studies are needed to confirm this conclusion.

## Introduction

Background

For many years, the proportion of procedures done in an outpatient setting has been rising quickly, and this trend is expected to continue. Children who have outpatient open inguinal surgery must get effective, short-lasting anesthesia with good intraoperative and postoperative analgesia to allow for an early discharge. However, children still experience a lot of prolonged post-operative pain [1]. Caudal epidural block and Ilioinguinal-Iliohypogastric (ILIH) nerve block techniques are commonly used for pain relief in pediatric patients. Many randomized controlled trials (RCTs) have shown that local anesthetic techniques are superior to systemic analgesics in children [1]. Using ultrasound (US) in regional anesthetics and pain treatment has grown [2]. In pain management and anesthetic therapy, ultrasound has improved the 'blind' traditional procedures of nerve blocks [3]. Although these blocks are typically secure and efficient, they may lead to several possible complications, such as infection if suitable sterile techniques are not used, systemic toxicity if local anesthetic is injected into a blood vessel, and nerve damage if US guidance is not used [4].

Caudal epidural block

In general, caudal epidural blocks have a high percentage of success, are comparatively safe, have a low risk of complications [4], and are frequently used for pediatric procedures. However, this is a central neuro-axial block and can lead to dangerous complications like the spread of local anesthetic into the subarachnoid space or extension of the block, giving rise to weakness of the lower limbs. For this block, the epidural space is approached through the sacral hiatus to deposit the local anesthetic drug in the caudal epidural space. US guidance can be used to locate the sacral hiatus, allowing needle entrance into the hiatus, and to visualize the progressing needle in the caudal canal. The US adds an advantage to the landmark-based approach in the pediatric population because the spinal cord and dural sac in children reach up to L3-4 and S3-4, respectively, with cephalad advancement occurring over the first year of life [2]. When doing a caudal epidural block on young infants, it is possible to penetrate the dural sac [4]. The sacral hiatus is typically easy to palpate in newborns and young children, making this approach considerably more straightforward and reliable [2]. The problems of caudal epidural block are like those that follow lumbar epidural nerve block, and they may involve both technique- and injectate-related issues (local anesthetic or other injected substance). Luckily, significant problems are rare. Cranial spread of local anaesthetics, epidural abscess, meningitis, epidural hematoma, dural puncture, post-dural puncture headache, subdural injection, pneumocephalus and air embolism, back discomfort, and damaged or twisted epidural catheters are rare but significantly dreaded side effects [2].

Ilioinguinal-iliohypogastric nerve block

The ilioinguinal-iliohypogastric block (ILIHB) is a well-known technique for postoperative analgesia; however, it is not very commonly used for open inguinal surgery in children. After passing inferiorly over the quadratus lumborum muscle, they pierce the aponeurosis of the transversus abdominis muscle near the lumbar triangle. They then travel between the transversus abdominis and internal oblique muscles, eventually entering the internal oblique approximately 1-3 cm medial to the anterior superior iliac spine. Ilioinguinal nerve was located approximately 1.9 ± 0.9 mm (left side) and 2.0 ± 0.7 mm (right side) from the anterior superior iliac spine (ASIS), while the iliohypogastric nerve was situated about 3.3 ± 0.8 mm (left side) and 3.9 ± 1.0 mm (right side) from the ASIS. With a wide variety of anatomical diversity, particularly concerning the innervation of the labia and scrotum, the ilioinguinal nerve supplies sensory innervation to the mons pubis and labia or scrotum, two parts of the groin. The genitofemoral nerve innervates in 40% of the cases. The iliohypogastric nerve gives the sensory innervation above the mons pubis and the groin. When US is employed, this block is remarkably efficient and secure [1]. Within the first 24 hours following surgery, US-ILIHB significantly decreased the occurrence of pain [5]. Patients with persistent inguinal post-herniorrhaphy discomfort can also benefit from using the block to treat their chronic pain [6].

Aim and rationale

This study aimed to evaluate and compare the analgesic effectiveness of two ultrasound-guided nerve blocks: caudal epidural and ILIH block for pediatric patients undergoing inguinal surgery. The rationale was to assess whether the less commonly used ILIH block, which is associated with fewer complications, could offer pain relief comparable to the more complex and frequently used caudal epidural block, which carries a higher risk of complications. The research question was: Is the ILIH block as effective as the caudal epidural block in providing postoperative analgesia in children undergoing inguinal surgery? The objective was to identify the more effective postoperative analgesic technique in children aged six months to 12 years undergoing inguinal procedures at Sultan Qaboos University Hospital. The primary outcome was pain relief and secondary outcomes were to calculate the rescue analgesia doses and the length of stay (block to ward discharge time).

## Materials and methods

Study design

This double-blinded RCT with two parallel arms was designed to compare the analgesic effectiveness of ultrasound-guided caudal with ilio-inguinal regional analgesia techniques in children undergoing open inguinal surgeries from December 2022 to November 2023, conducted at the Department of Anesthesia and Intensive Care, Sultan Qaboos University Hospital, Muscat, Oman.

Participants

Inclusion criteria were American Society of Anesthesiologists (ASA) I and II patients aged six months to 12 years with elective open-inguinal procedures. Exclusion criteria were refusal of consent, ASA III and higher-risk group, hemodynamic instability, local infection over the insertion site, coagulopathy, known allergy to local anesthetic medications, and complex anatomy.

Ethical consideration

Ethical approval for this study was obtained from the Medical Research Ethics Committee (MREC#2919) on November 16, 2022, and it was registered in the Clinical Trials Registry (NCT05558748). Patient recruitment started after the registration. An appropriate population of patients was approached and randomized. The procedure and the study details were explained to the parents/surrogates using an information sheet produced in the local language as well as English. If they agreed to participate, informed consent was obtained from the parent.

Interventions

Participants were assigned randomly to two groups: Group 1 for a caudal epidural block with US guidance and Group 2 for an ILIH nerve block with US guidance. The study and its salient features were explained to the parents, or patient surrogates, with the help of an information sheet containing all the study details. Once they agreed, informed consent was obtained. Pre-anesthesia check-up was done by a separate anesthetist as routine, and patients were prescribed premedication (oral midazolam 0.3 mg/kg) 30 minutes prior to shifting the patient to the operating room. After entering the operating room, the ASA monitors were connected (heart rate, ECG, respiratory rate, oxygen saturation, non-invasive blood pressure, and temperature), and anesthesia was performed by one of the two trained pediatric anesthetists. Propofol (2.5-3.5 mg/kg) was used for induction, and a second-generation supraglottic airway device (Pro-Seal laryngeal mask airway) was inserted to maintain the airway. Sevoflurane was administered to maintain anesthesia. The same anesthetist performed the block in line with randomization. Granestron (0.01 mg/kg) and dexamethasone (0.15 mg/kg) were administered for postoperative nausea and vomiting prophylaxis 10 minutes after administering the block. Paracetamol was administered intravenously at a dose of 7.5 mg/kg for babies under 10 kg and 15 mg/kg for babies beyond 10 kg immediately after the block for multimodal intraoperative analgesia. Follow-up assessments were performed in PACU (0, 30 minutes and at exit from PACU), postoperatively in the ward at two, three, and six hours, and at the time of discharge from the ward (daycare surgeries) using FLACC scale and at home on day two and day seven using the numeric rating scale (NRS). In case of pain, the rescue analgesia was given with fentanyl 0.5 mcg/kg aliquots in the operating room and PACU and with syrup paracetamol (10 mg/kg orally) in the ward. On days two and seven at home, the patients were evaluated telephonically regarding pain using the NRS, analgesic use, return to daily activities, and any side effects like reduction in muscle power or swelling, etc. For rescue analgesia in the ward or at home, syrup paracetamol (10 mg/kg) was prescribed.

*Caudal Epidural Block* 

After anesthesia induction, the patient was turned to the lateral position, and the sacral cornua was palpated using the anatomic landmarks as a guide. Both the area and the skin were fully draped and sterile. A high-frequency linear array probe was positioned in the transverse plane over the two sacral cornua, represented by two symmetric hyperechoic arches connected by a hypoechoic shadow that lay beneath both lines. Initial scanning in the transverse plane allowed visualization of the midline and identification of the sacrococcygeal ligament between the two sacral cornua. The two cornua resemble the two eyes of a frog, called the frog-eye sign. The probe was then rotated 90 degrees for a longitudinal view, and the needle was advanced at a 20-degree angle with visualisation of the needle tip and length to puncture the area between the two cornua. A noticeable “pop” was felt when the needle pierced the sacrococcygeal ligament. The drug was injected into the caudal space when the needle tip reached it. This was done under ultrasonography guidance while always maintaining the tip visible. Levo-bupivacaine 0.25%, 1 ml/kg, was administered in the caudal space.

ILIHB

The patient was maintained in a supine position. The high-frequency linear array probe was positioned on the anterior abdominal wall in the plane that connected the umbilicus to the anterior superior iliac spine. Both nerves were observed as hypoechoic structures surrounded by hyperechoic tissue near the ASIS between the transversus abdominis and internal oblique muscles. Inserting the needle tip towards the block plane was obtained from medial to lateral following the in-plane technique. Levo-bupivacaine (0.25%) at a dose of 0.4 ml/kg was injected gradually into the fascial plane between the internal oblique and transversus abdominis plane, encircling the nerves.

Outcomes

The primary outcome was degree of pain relief. The secondary outcomes were use of rescue analgesia postoperatively and the postoperative length of stay.

Sample size

The estimated sample size was 128 (64 in each group). The estimated effect size (medium, Cohen's d=0.5) for the variation in the post-operative pain score between the caudal and ilioinguinal block techniques served as the basis for the estimation. The alpha error was set to 5% and the power to 80%. There was a 1:1 allocation ratio. G Power 3.1.9.7 was the software used to calculate the sample size. However, the open surgical technique was discontinued, so the study was prematurely stopped at 20 patients.

Randomization and blinding

The randomization sequence was generated by a remote computer by the statistics department and was available in sequentially numbered sealed envelopes with the principal investigator. The randomization was done at the time of identification of eligible patients. The principal investigator communicated the randomization group to the block performing anesthetist just at the start of anesthesia opening by the individual envelope. After surgery, when the patient was transferred to PACU and then the ward, none of the assessors knew the group the patient belonged to. In this trial, the patient and the postoperative assessor were blinded to the type of nerve block received.

To prevent bias, the whole team, including anesthetists, assessors, and analyzers, strictly followed the anesthesia management protocol with nerve block. All the investigators completed a good clinical practice (GCP) course to qualify for RCT participation. Two well-trained anesthetists with excellent expertise in ultrasound-guided nerve blocks were chosen to perform the blocks. The postoperative pain assessor used well-validated and commonly used pain scores to assess the pain. The blinding was well maintained at two specified levels.

Statistical methods

SPSS version 25 (IBM Corp., Armonk, NY, USA) and STATA SE 17 (StataCorp., College Station, TX, USA) were used for data analysis and projection. Normality was checked for the continuous variables, and they were reported as Mean+/-SD or Median, interquartile range (IQR) according to their distribution. The categorical variables were reported as Numbers (%) and analysis was done with the Fisher Exact test. The significance was analyzed with the Friedman test for the FLACC score postoperatively in the hospital. The Mann-Whitney U test was used for NRS scores postoperatively at home. The postoperative length of stay (Block to Ward discharge time) was analyzed using the Mann-Whitney U test. Postoperative rescue analgesia was projected and analysed using a Kaplan-Meier graph and log-rank test. P value <0.05 was considered as significant. 

## Results

**Figure 1:**
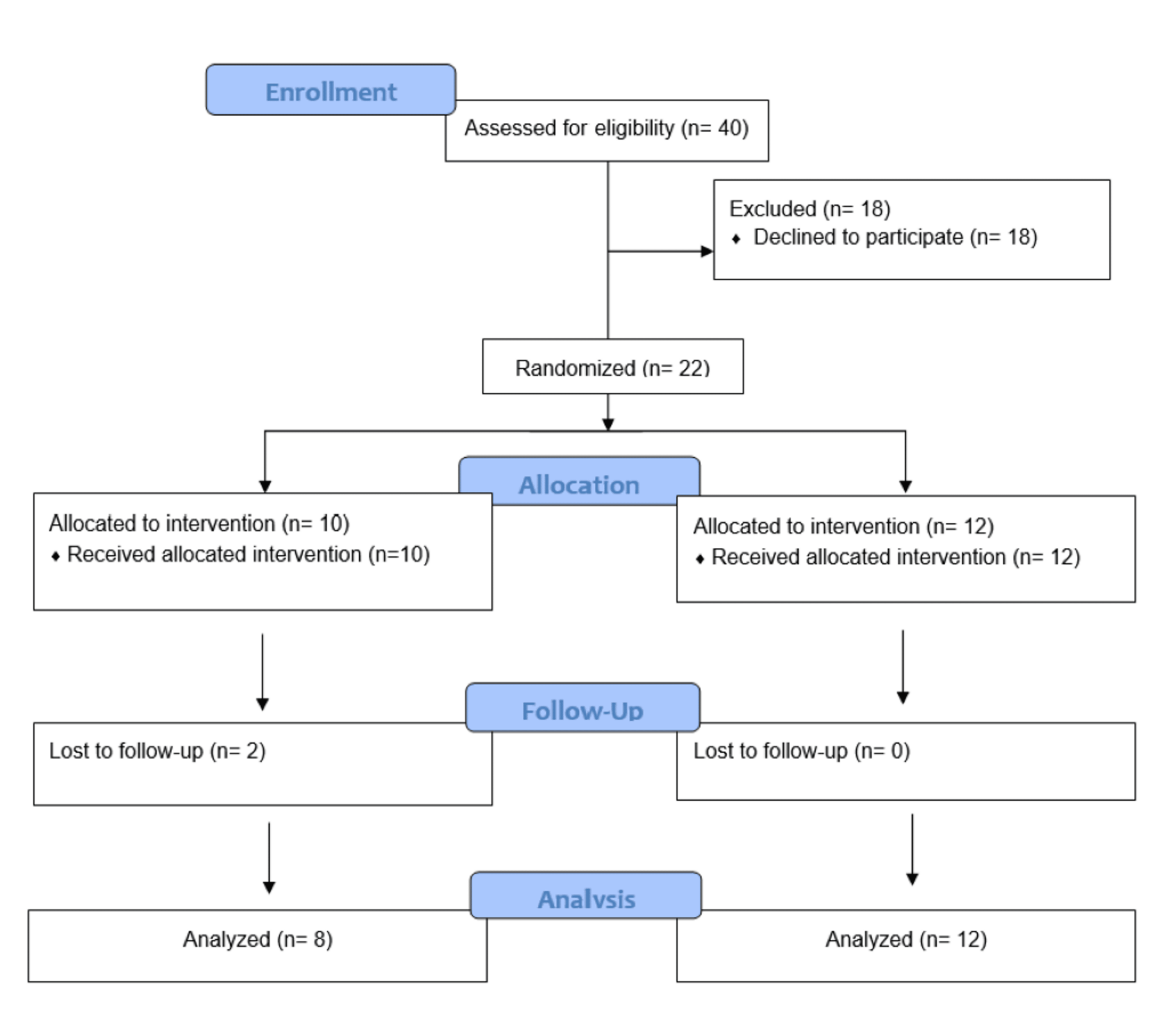
Consolidated Standards of Reporting Trials (CONSORT) diagram (patient flow across the study)

During the study period from December 2022 to November 2023, 40 patients were approached, but only 22 patients consented and could be included in this study. In the randomization groups, 10 patients received the caudal epidural block (Group 1), while 12 received the ilioinguinal nerve block (Group 2). Two patients in Group 1 were lost to follow-up, in contrast to no loss in Group 2. Eventually, eight patients from Group 1 and 12 from Group 2 were included in the analysis (Figure 1).

The study was stopped as the surgeons changed their surgical approach from open to laparoscopy.

The two groups had similar demographic and intervention characteristics (Table 1).

**Table 1:** Patient demographics. Group 1: Caudal block; Group 2: Ilioinguinal\Iliohypogastric block; SD: Standard deviation; ASA: American Society of Anesthesiologist; FLACC Score: Face, Legs, Activity, Cry, and Consolability Score; PACU: Post-Anesthesia Care Unit; NRS: Numeric Rating Scale; LAST: Local Anaesthetic Systematic Toxicity, * Chi-Square Test, **Fisher Exact Test, *** Mann-Whitney U Test

Variables		Group 1 (n=8)	Group 2 (n=12)	Significance	Group 1+2 (n=20)
Age [Mean (SD)]		2.13 (1.356)	3.67 (1.303)	0.030***	3.05 (1.504)
Weight [Mean (SD)]		11.3 (2.475)	15.92 (4.420)	0.010***	14 (4.401)
ASA Grade [n (%)]	I	6/8 (75)	12/12 (100)		18/20 (90)
	II	2/8 (25)		0.211**	2/20 (10)
Surgery	Inguinal herniotomy	5/8 (62.5)	10/12 (83.3)		15/20 (75)
	Orchidopexy	3/8 (37.5)	2/12 (16.7)	0.628**	5/20 (25)
FLACC Score [Mean (SD), Median (IQR) ]	At the end of anesthesia (time 0)	0 (0), 0 (0-0)	0 (0), 0 (0-0)	-	0 (0), 0 (0-0)
	In PACU (30 min after time 0)	1.75 (2.435), 0(0-4.5)	1.25 (1.658), 2 (0-3.0)	0.590*	1.45 (1.959), 0 (0-3)
	At discharge from PACU	0.50 (.756), 0.5 (0-1.00)	0.08 (0.289), 0 (0-0.25)	0.098*	0.25 (.550), 0 (0-1)
	At 2 hours from time 0	0 (0), 0 (0-0)	0.58 (.900), 0 (0-2.0)	0.086	0.35 (.745)
	At 3 hours from time 0	0 (0), 0 (0-0)	0 (0), 0 (0-0)	-	0 (0), 0 (0-0)
	At 6 hours from time 0	0 (0), 0 (0-0)	0 (0), 0 (0-0)	-	0 (0), 0 (0-0)
	At discharge from the ward	0 (0), 0 (0-0)	0 (0), 0 (0-0)	-	0 (0), 0 (0-0)
NRS*[Mean (SD), Median (IQR)]	D2	0.25 (.707), 0 (0-0.5)	0.33 (.778), 0 (0-2.0)	0.626	0.30 (.733), 0 (0-1)
	D7	0 (0), 0 (0-0)	0 (0), 0 (0-0)	-	0 (0), 0 (0-0)
Rescue Analgesia [n(%)]	0	3/8 (37.5)	8/12 (66.7)	-	11/20 (55)
	1	3/8 (37.5)	3/12 (25)	-	6/20 (30)
	2	2/8 (25)		-	2/20 (10)
	3	1/12 (8.3)	1/20 (5)	0.362*	1/20 (5)
Day of Resumption of Full Activity [Mean (SD)]		0.50 (.535)	0.83 (1.403)	0.431***	0.70 (1.129)
Muscle Power [n (%)]	Reduced	0/8 (0)	0/12 (0)	-	0/20 (0)
	Normal	8/8 (0)	8/12 (0)	-	20/20 (0)
Complications [n (%)]	Failed block	0/8 (0)	0/12 (0)	-	0/20 (0)
	Local bleeding	0/8 (0)	0/12 (0)	-	0/20 (0)
	LAST	0/8 (0)	0/12 (0)	-	0/20 (0)
	Urinary retention	0/8 (0)	0/12 (0)	-	0/20 (0)
	Prolonged block recovery	0/8 (0)	0/12 (0)	-	0/20 (0)
Block to Ward Discharge Time [Mean (SD)]		283 (102.182)	309.83 (79.409)	0.746***	299.1 (87.63195)

The mean age of Group 1 was 2.13 (1.356), while that of Group 2 was 3.67 (1.303) (p=0.030). Overall, 15 (75%) patients underwent inguinal herniotomy, and five underwent orchidopexy (25% of all) (p=0.628). Most patients (90%) were ASA grade one, and the remaining (10%) were ASA grade two (p=0.211). Only two patients (10%) had comorbidities and belonged to Group 1 (asthma and sickle cell trait). 

**Figure 2:**
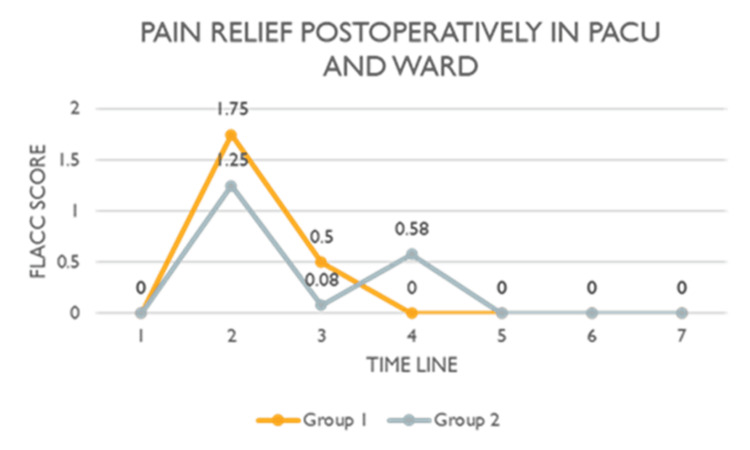
Postoperative FLACC scores across the two groups. PACU: Post-Anesthesia Care Unit; FLACC Score: Face, Legs, Activity, Cry, and Consolability Score; Group 1: Caudal Block; Group 2: Ilioinguinal/Iliohypogastric Block. The projected pain scores at different time points were analyzed using Friedman test.

The primary outcome was the degree of pain relief. There was no significant difference between the two groups regarding the FLACC pain score 30 minutes after arrival (p=0.590) and before discharge from PACU (0.098), postoperatively in the ward at two (p=0.086), three, and six hours, and at the time of discharge from the ward (Table 1, Figure 2).

**Figure 3:**
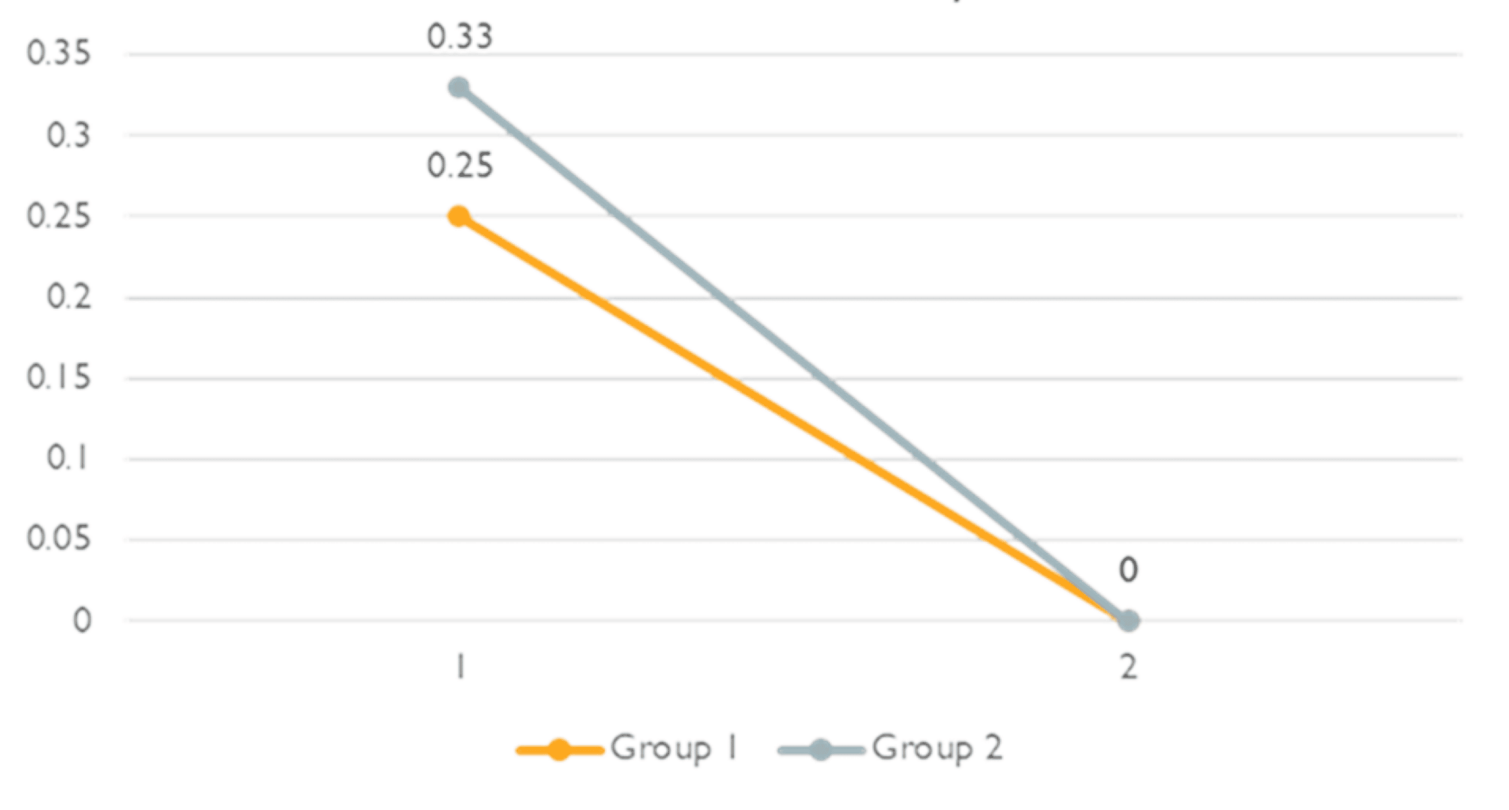
NRS scale at home on days two and seven. NRS: Numeric Rating Scale; Group 1: Caudal Block; Group 2: Ilioinguinal/Iliohypogastric Block. The projected scores at different time points are analyzed with the Mann-Whitney U Test.

Mann-Whitney U test showed no significant difference in the degree of pain relief postoperatively in the ward (p=0.792). Moreover, the difference between the two groups regarding the NRS scale at home on days two and seven was also insignificant (Figure 3).

**Figure 4:**
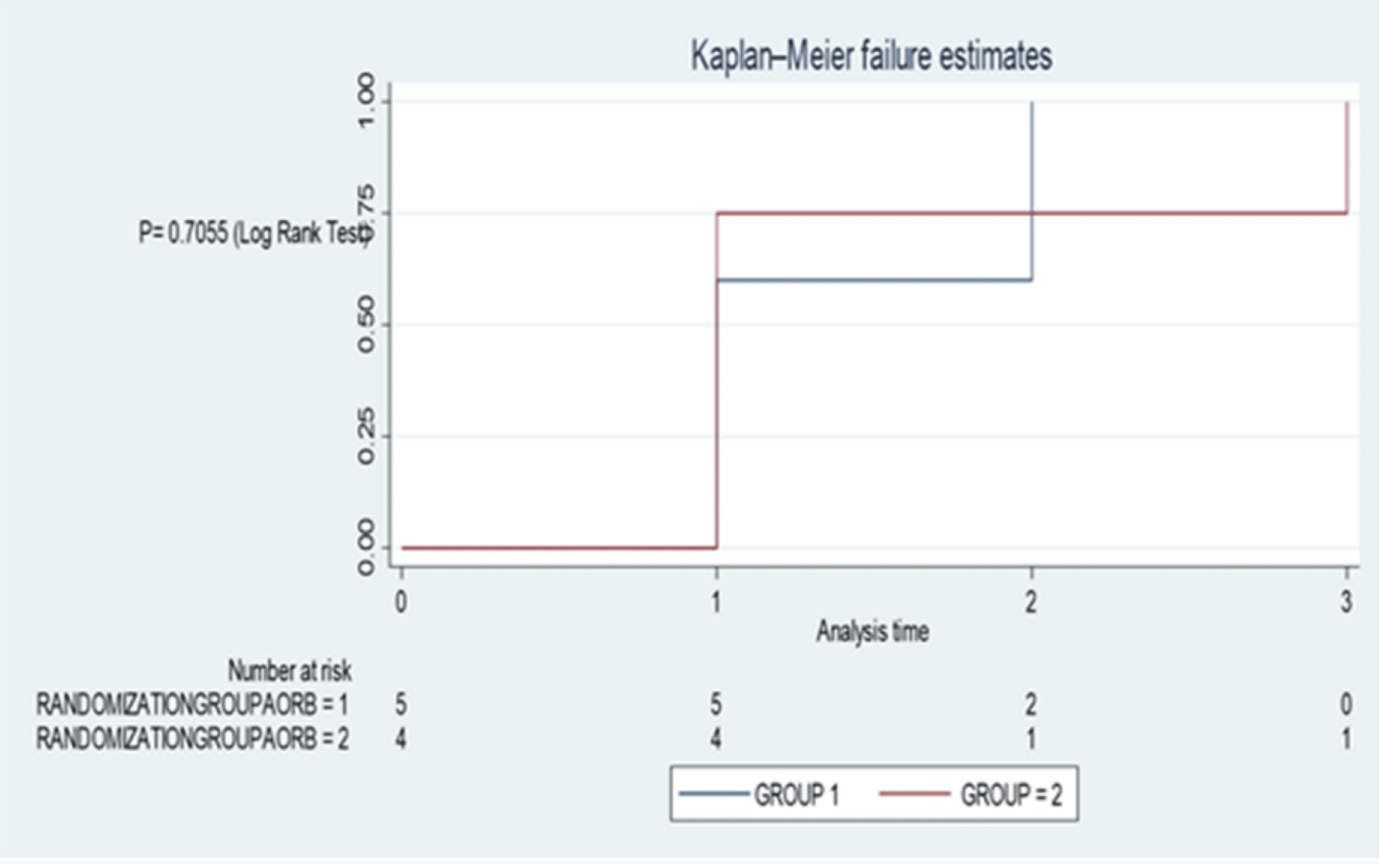
Use of rescue analgesia in hospitals for both groups. Group 1: Caudal Block; Group 2: Ilioinguinal/Iliohypogastric Block. P-value is estimated using the Log-Rank Test with Kaplan-Meier Estimate. This is a Kaplan–Meier failure curve, showing the cumulative probability of requiring rescue analgesia over time (i.e., the “failure” = event of needing rescue analgesia). The x-axis represents postoperative time; the y-axis shows cumulative failure probability, i.e., proportion of patients who required rescue analgesia. Both lines stay at 0 initially, meaning no patients required rescue analgesia immediately after surgery. Then, events (failures) occurred over time (seen as steps upward on the curves). Eventually, the curves appear quite close together and show similar trajectories; P = 0.7055. Log-rank test: A p-value > 0.05 means no statistically significant difference between the groups in terms of time to first rescue analgesia. In other words, both blocks were equally effective in avoiding or delaying the need for rescue analgesia in hospital. This suggests comparable analgesic effectiveness of the two regional techniques in the immediate postoperative period.

The secondary outcomes were the reduction in rescue analgesia postoperatively at home and postoperative length of stay. There was no difference in the rescue analgesia given in the hospital for both groups (p=0.7055, log-rank test by Kaplan-Meier failure estimation) (Figure 4).

**Figure 5:**
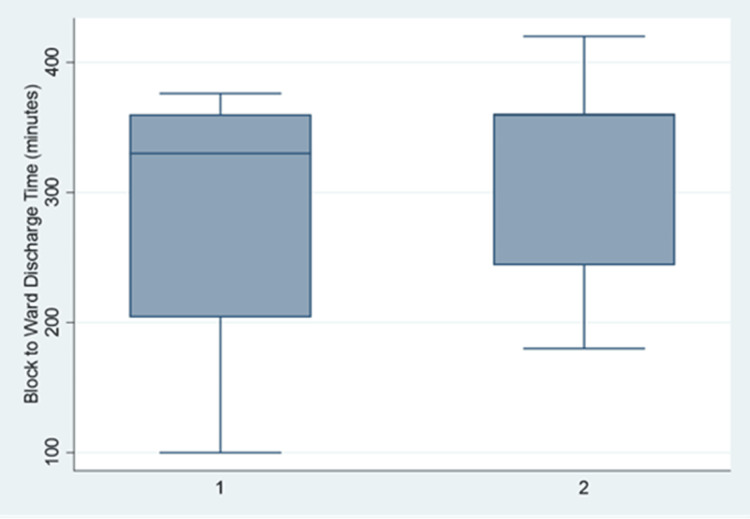
Postoperative length of stay (block-to-ward discharge time) across the two groups. Group 1: Caudal Block; Group 2: Ilioinguinal/Iliohypogastric Block. Block to ward discharge time of 283 minutes versus 309.83 minutes (Group 1 versus Group 2) was analyzed using Mann-Whitney U test, p=0.746

Moreover, the two groups had no significant difference regarding block-to-ward discharge time (p=0.746, Mann-Whitney U Test) (Figure 5).

Postoperative rescue analgesia was required by four of 12 (33.3%) patients in the ILIH block group and five of eight (62.5%) patients in the caudal epidural block group during their hospital stay or at home (p=0.199, Fisher Exact Test).

## Discussion

This study explored the analgesic effectiveness of caudal analgesia to ilioinguinal regional analgesia techniques in children undergoing inguinal surgeries with US guidance and found both the blocks to be equally effective (in terms of analgesia and length of stay) and with low complications.

The caudal epidural block is trendy and commonly used for pediatric inguinal surgeries. Still, it has a high chance of dreaded complications, such as hypotension, a cephalic extension of the block, neural infections, prolonged muscle weakness, and bleeding [2]. However, the ilioinguinal nerve block is easy to perform, effective, and has a low complication rate (bleeding and infections). Still, it is not popular for pediatric inguinal surgeries in this part of the world.

We performed this study with ultrasound guidance, which imparts high success rates and is the recent way of performing blocks. Similarly, a prospective, crossover, randomized controlled study in 2012 was conducted on children having surgery on their groin alone. The trial comprised 50 children aged one to six years who were scheduled for unilateral groin surgery, and revealed that ILIHB with ultrasound guidance is an ideal intervention for hernia repair in children as effective as caudal epidural block [7]. A prospective randomized, double-blind study by Willschke et al. in 2005 evaluated the effectiveness of ultrasonography versus the traditional ILIHB approach [8]. The study comprised one hundred children (aged one month to eight years) who were scheduled for hydrocele surgery, orchidopexy, or inguinal hernia repair. It showed that ultrasound-guided IL/IH is more effective than a traditional one.

Our study showed that the mean FLACC score at discharge from the ward was zero for both groups. This outcome was comparable with a study done by Yimer et al. in 2020 in which they did the observations at discharge from the PACU and at the first, second, fourth, sixth, and 12th postoperative hours (p>0.05) [9]. Both showed similar effectiveness levels when using caudal and ILIHB.

Based on the results mentioned above about the NRS scale on days two and seven, we concluded that the pain gradually decreased without complications (Figure 3). Moreover, the length of stay, measured by block-to-ward discharge time, was almost similar with ILIH 309.83 (79.409) and caudal epidural block 283.00 (102.182) (p=0.792).

Overall, there was a considerable difference between the two groups concerning the requirement of postoperative rescue analgesia throughout the hospital admission or at home (5/8(62.5%) patients in the caudal group and 4/12(33.3%) patients in the ilioinguinal group) (p=0.199). This result favors the ilioinguinal group. However, this cannot be concluded because of inadequate numbers. However, we can presume or make a hypothesis for a more extensive study to prove this point. Our finding is similar to that of another study done in 2009 by Jagannathan et al., who found a higher requirement for rescue analgesics with the caudal block group compared to the ilioinguinal group during their hospital stays [10].

Regarding the complications and muscle weakness postoperatively, none of the patients had any decreasing muscle power or side effects in caudal and ILIH. Our result aligned with two studies by Bhattarai et al. in 2005 and Seyedhejazi et al. [11,12]. In contrast, another prospective cohort study was carried out involving 70 patients who underwent general anesthesia for inguinal operations. Patients in the IL/IH group (n=35) were compared with the caudal block group (n=35), and it was observed that some patients developed weakness in the leg due to high doses of local anesthetics in both blocks (1 ml/kg of 0.25% bupivacaine for caudal block and 0.4 ml/kg of 0.25% bupivacaine for IL/IH block). In the IL/IH group, there were two patients (5.7%) with leg weakness compared to four (11.4%) in the caudal group. This difference was statistically insignificant (p=0.39) [9].

Strengths and limitations

The study employs a double-blind, randomized controlled design, which minimizes bias and enhances the reliability of the findings. The use of ultrasound-guided techniques is a notable strength, as it ensures precision and reduces complications. The involvement of well-trained anesthetists and adherence to GCP guidelines reflect a high standard of execution. In terms of novelty, the study addresses a gap in research by comparing two analgesic techniques in the pediatric population, particularly in Oman, where such studies are scarce.

The small sample size (20 patients) is a significant limitation, as it restricts the generalizability and statistical power of the findings. Conducting the study in a single center limits its applicability to other settings and populations. While the study notes differences in the need for rescue analgesia, the statistical insignificance (p=0.199) weakens the argument for one technique over the other. In addition, the absence of reported complications may be due to the limited sample size, which might not capture rarer adverse events.

As for generalizability, this study involves a common surgical procedure, and both blocks are well-known and indicated. Hence, this study can be replicated easily, and the results can be applied to any population.

## Conclusions

In this exploratory study, the ilioinguinal nerve block was as effective as the caudal block in pain relief for open inguinal surgeries in the pediatric population. There was a considerable difference in the need for rescue analgesia in favor of ilioinguinal block, but the complications and length of stay were similar between the two block groups. However, we cannot conclude anything as the sample size was limited. These results should prompt the generation of a hypothesis for a more extensive study, as this is an everyday issue wherever an open surgical technique is used for pediatric inguinal surgeries. A simple and more superficial block could suffice.

## References

[1] Grosse B, Eberbach S, Pinnschmidt HO, Vincent D, Schmidt-Niemann M, Reinshagen K (2020). Ultrasound-guided ilioinguinal-iliohypogastric block (ILIHB) or perifocal wound infiltration (PWI) in children: a prospective randomized comparison of analgesia quality, a pilot study. BMC Anesthesiol.

[2] NYSORA (2019 (2023). Caudal anesthesia. https://www.nysora.com/techniques/neuraxial-and-perineuraxial-techniques/caudal-anesthesia/.

[3] Eichenberger U, Greher M, Kirchmair L, Curatolo M, Moriggl B (2006). Ultrasound-guided blocks of the ilioinguinal and iliohypogastric nerve: accuracy of a selective new technique confirmed by anatomical dissection. Br J Anaesth.

[4] Dua A, Afzal M (2025). Caudal anesthesia. StatPearls [Internet].

[5] Karim WA, Bathla S, Malik S, Arora D (2020). Comparison of ultrasoundguided ilioinguinal iliohypogastric nerve block with wound infiltration during pediatric herniotomy surgeries. Anesth Essays Res.

[6] Ilioinguinal/iliohypogastric block (2023 (2023). Ilioinguinal/iliohypogastric block. https://www.euroespa.com/science-education/specialized-sections/espa-pain-committee/us-regional-anaesthesia/truncal-blocks/ilioinguinaliliohypogastric-block/.

[7] Abdellatif AA (2012). Ultrasound-guided ilioinguinal/iliohypogastric nerve blocks versus caudal block for postoperative analgesia in children undergoing unilateral groin surgery. Saudi J Anaesth.

[8] Willschke H, Marhofer P, Bösenberg A (2005). Ultrasonography for ilioinguinal/iliohypogastric nerve blocks in children. Br J Anaesth.

[9] Yimer Y, Mohammed A, Ali S (2019). Analgesic effect of caudal and IL/IH nerve blockade among children undergoing inguinal surgeries: a prospective cohort study. Int J Surg Open.

[10] Jagannathan N, Sohn L, Sawardekar A (2009). Unilateral groin surgery in children: will the addition of an ultrasound-guided ilioinguinal nerve block enhance the duration of analgesia of a single-shot caudal block?. Paediatr Anaesth.

[11] Bhattarai BK, Rahman TR, Sah BP, Tuladhar UR (2005). Analgesia after inguinal herniotomy in children: combination of simplified (single puncture) ilioinguinal and iliohypogastric nerve blocks and wound infiltration vs. caudal block with 0.25% bupivacaine. Kathmandu Univ Med J (KUMJ).

[12] Seyedhejazi M, Sheikhzadeh D, Adrang Z, Rashed FK (2014). Comparing the analgesic effect of caudal and ilioinguinal iliohypogastric nerve blockade using bupivacaine-clonidine in inguinal surgeries in children 2-7 years old. Afr J Paediatr Surg.

